# Characterization of the complete chloroplast genome of *Quercus virginiana* Mill. (Fagaceae)

**DOI:** 10.1080/23802359.2021.1886004

**Published:** 2021-03-15

**Authors:** Dan Liu, Wen-Qing Li, Xiao-Man Xie, De-Shen Liu, Feng Li, Guangchen Gao, Zhen-Jie Zhuang, Yi-Zeng Lu, Wei Li

**Affiliations:** a College of Biological Sciences and Biotechnology, Beijing Forestry University, Beijing, People’s Republic of China; b Shandong Forest Germplasm Resources Center, Jinan, People’s Republic of China; c Yishan State owned Forest Farm of Yishui County, Linyi, People’s Republic of China

**Keywords:** *Quercus virginiana*, chloroplast genome, phylogenetic analysis

## Abstract

The complete chloroplast genome of *Quercus virginiana* was sequenced with Illumina HiSeq 2000 platform. It was a typical quadruple structure as other plants of *Quercus* with 161,221 bp in length, including a large single-copy (LSC: 90,553 bp) region and a small single-copy (SSC: 19,016 bp) which were separated by a pair of inverted repeats (IRa, b: 25,826 bp) region. The overall GC content is 36.9%. A total of 131 genes was annotated which contained 86 protein-coding genes including the Trans splicing gene of *rps*12, 37 tRNA genes, and 8 rRNA genes. ML phylogenetic analysis compared with 17 expressed chloroplast genomes revealed that *Q. virginiana* was a sister to other species of *Quercus*, which were grouped together with five species of Section *Quercus* and another 12 species of *Quercus* were divided into another group.

*Quercus virginiana* Mill. is a multipurpose tree species of *Quercus* in Fagaceae, one of the commonest and best known species in the coastal region of the southeastern United States. Which was the strategic resource tree of the United States, because of it’s the hardest, strongest and toughest wood of all oaks (Elias 1971). It is also an excellent evergreen broad-leaved tree species in beach wetland, saline alkali land greening and coastal windbreak forest construction (Harms 1990)*. Quercus virginiana* has been widely and long-term introduced in the Yangtze River Delta of China (Chen et al, 2007). And the characteristics of adaptability of introduced species, such as cold resistance (Zhang et al. 2018), light adaptability (Wang et al. 2019) and mycorrhizal types (Jin et al. 2020) have been revealed at present. However, the genomic information of *Q. virginiana* lacked that would be helpful to the study of scientific introduction and efficient utilization.

The fresh leaves of *Q. virginiana* were collected from the living individual permanently conserved in the *Quercus* gene bank (36.77°N, 117.471°E) which was introduced and bred in 2014. The specimens were preserved in the herbarium of Shandong Forest Germplasm Resources Center (barcode SDF2019L0012). Total genomic DNA (saved in DNA library of Shandong Forest Germplasm Resources Center with the code of fjnyl2020cp03) was extracted by the Plant DNA extraction Kit (TIANGEN, Beijing, China) according to the requirements of the reagent company.

Paired-end reads were constructed according to the Illumina library preparation protocol and sequenced on an Illumina HiSeq 2000 platform. The whole chloroplast genome of *Q. virginiana* was assembled by MITObim v1.8 (Hahn et al. 2013) and was annotated in DOGMA (http://dogma.ccbb.utexas.edu/). The whole chloroplast genome of *Q. virginiana* and other 17 published plastomes of *Quercus* were conducted by using MAFFT v7.429 (Katoh and Standley 2013), with *Juglans cathayensis* as outgroup. Maximum-likelihood (ML) phylogenetic tree with 1000 bootstrap replicates was inferred using IQ-TREE v1.6.12 (Lam-Tung et al. 2015) and TVM + F+R2 model.

The chloroplast genome of *Q. virginiana* (GenBank accession number MT916773) was also a typical quadruple structure with 161,221 bp in length that contains a large single-copy (LSC: 90,553 bp) region and a small single-copy (SSC: 19,016 bp), which were separated by a pair of inverted repeats (IRa, b: 25,826 bp) region. The overall GC content was 36.9%. A total of 131 genes were annotated, including 86 protein-coding genes, 37 tRNA genes, and 8 rRNA genes. Among these, 15 genes had a single intron respectively, *clp*P and *ycf*3 had two introns respectively. While *rps*12 had Trans splicing function. The chloroplast genome of *Q. virginiana* was similar to that of *Q. aliena*, *Q. dentate*, *Q. wutaishanica*, *Q. mongolica* and *Q. robur*, which were grouped together as the member of the Section *Quercus*. While the other 12 species of *Quercus* were divided into another group. ML phylogenetic analysis compared with 17 expressed chloroplast genomes of *Quercus* revealed that *Q. virginiana* was a sister to other *Quercus* species (Figure 1).

**Figure 1.**
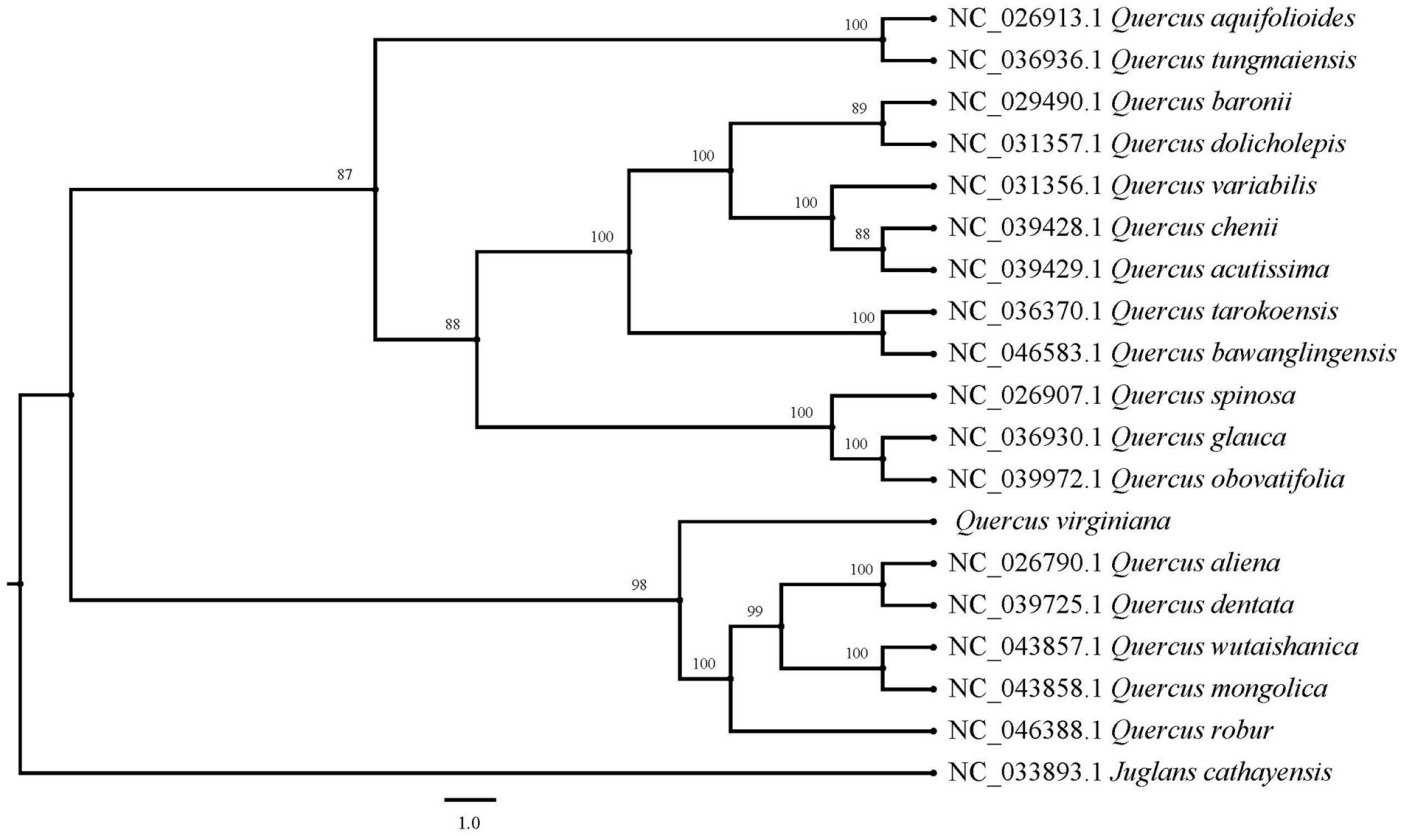
A maximum-likelihood (ML) tree of *Q. virginiana* and other 17 related species of *Quercus* based on the complete chloroplast genome sequences with *J. cathayensis* as outgroup. The accession numbers are showed in the figure, and the numbers behind each node are bootstrap support values.

## Data Availability

The genome sequence data that support the findings of this study are openly available in GenBank of NCBI at (https://www.ncbi.nlm.nih.gov/) under the accession no. MT 916773. The associated BioProject, SRA, and Bio-Sample numbers are PRJNA694782, SRR13528289, and SAMN17574357 respectively.
